# Prevalence and factors associated with family planning during COVID-19 pandemic in Bangladesh: A cross-sectional study

**DOI:** 10.1371/journal.pone.0257634

**Published:** 2021-09-21

**Authors:** Nitai Roy, Md. Bony Amin, Maskura Jahan Maliha, Bibhuti Sarker, Md Aktarujjaman, Ekhtear Hossain, Gourango Talukdar

**Affiliations:** 1 Department of Biochemistry and Food Analysis, Patuakhali Science and Technology University, Patuakhali, Bangladesh; 2 Faculty of Nutrition and Food Science, Patuakhali Science and Technology University, Patuakhali, Bangladesh; 3 Department of Economics, University of Manitoba, Winnipeg, Canada; 4 Department of Economics, Bangabandhu Sheikh Mujibur Rahman Science and Technology University, Gopalganj, Bangladesh; 5 Department of Biological Sciences and Chemistry, Southern University and A&M College, Baton Rouge, LA, United States of America; 6 Department of Neuroscience, University of Minnesota, Minneapolis, MN, United States of America; Shahjalal University of Science and Technology, BANGLADESH

## Abstract

**Background and objectives:**

The COVID-19 pandemic has negatively impacted health systems worldwide, including in Bangladesh, limiting access to family planning information (FP) and services. Unfortunately, the evidence on the factors linked to such disruption is limited, and no study has addressed the link among Bangladeshis. This study aimed to examine the socioeconomic, demographic, and other critical factors linked to the use of FP in the studied areas during the COVID-19 pandemic.

**Methods:**

The characteristics of the respondents were assessed using a cross-sectional questionnaire survey and descriptive statistics. The variables that were substantially linked with FP usage were identified using a Chi-square test. In addition, a multivariate logistic regression model was used to identify the parameters linked to FP in the study areas during the COVID-19 pandemic.

**Results:**

The prevalence of FP use among currently married 15–49 years aged women was 36.03% suggesting a 23% (approximately) decrease compared to before pandemic data. Results also showed that 24.42% of the respondents were using oral contraceptive pills (OCP) which is lower than before pandemic data (61.7%). Multivariate regression analysis provided broader insight into the factors affecting FP use. Results showed that woman’s age, education level of the respondents, working status of the household head, locality, reading a newspaper, FP workers’ advice, currently using OCP, ever used OCP, husbands’ supportive attitude towards OCP use, duration of the marriage, ever pregnant, the number of children and dead child were significantly associated with FP use in the study areas during COVID-19 pandemic.

**Conclusions:**

This study discusses unobserved factors that contributed to a reduction in FP use and identifies impediments to FP use in Bangladesh during the COVID-19 epidemic. This research further adds to our understanding of FP usage by revealing the scope of the COVID-19 pandemic’s impact on FP use in Bangladesh’s rural and urban areas.

## 1. Introduction

The World Health Organization (WHO) declared the outbreak of SARS-COV-2 (Severe Acute Respiratory Syndrome–related coronavirus-2) to be a public health emergency of international concern on January 31, 2020 [1]. Bangladesh declared the first diagnosed case of COVID-19 infection on March 8, 2020 [2], and the number of cases has been rising since then. Infectious outbreaks may potentially devastate some regular medical services, such as family planning (FP) programs [3]. For instance, practice of contraception declined by 65% in Liberia and 23% in Sierra Leone during the West African Ebola epidemic [3]. Most countries across the globe have recognized this as a national emergency and have started taking measures against the infection accordingly [4]. As a result, countries will need to coordinate their strategies in order to keep the crucial healthcare system running during this crisis.

Bangladesh is one of the most densely populated countries in the world [5]. Its government viewed rapid population growth as a high priority problem, and decided to launch the national FP program in the mid-1970s. Since then, government of Bangladesh (GOB) has attempted to strengthen the FP program through increased resource allocation, multi-sectoral collaboration, mass media campaigns, employing field-staff to provide domiciliary FP services, and involving voluntary and private agencies. The government has been successful, to some extent, even in the context of a Muslim-majority country characterized by higher poverty, a lower literacy rate, and a lower level of women’s autonomy [6–8].

However, the FP program in Bangladesh–which was once considered the role model for developing countries–faces daunting challenges during COVID-19 pandemic. The international planned parenthood federation (IPPF) report showed that 1872 clinics and other outlets were closed in the South Asian region because of this pandemic [9]. Another report of UNICEF showed that around 2.4 million babies and pregnant mothers in Bangladesh would die during this COVID-19 pandemic, while the global number is 116 million [9]. This statistics indicate the importance of FP use during the COVID-19 pandemic.

COVID-19 is affecting women’s ability to use contraception in several ways. First, disruptions to the supply chain are limiting the production, distribution, and availability of contraceptive commodities, resulting in stock-outs [10]. Second, some healthcare facilities are reducing services [11], and FP workers are being redirected from providing FP services to other emergency services in response to the COVID-19 pandemic. Foreign policy, an online-based news portal, published a news article on May 13, 2020 showing that the Coronavirus disease is upending family planning efforts in India [12]. The news article also reported that India, Nepal, Uganda, and Zimbabwe face increased unplanned pregnancies as contraception access disappears. In particular, the COVID-19 pandemic has resulted in a 10% decline in the use of short- and long-acting reversible contraception in low- and middle-income countries, which resulted in an additional 15 million unintended pregnancies over the year. Third, many women can not visit healthcare facilities due to strict lockdowns measures or fear of exposure to COVID-19 [13].

Although national surveys have extensively looked at measures and changes in FP programs in Bangladesh, to the best of our knowledge, little is known about it among Bangladeshi women during the COVID-19 pandemic. This study thus aims at exploring knowledge and practices concerning FP use and birth spacing, perceptions about health care quality, and barriers and facilitators that affect FP use during the COVID-19 pandemic.

## 2. Methodology

### 2.1 Data source and sample

We followed a purposive sampling method to collect primary data. In our purposive sampling, subjects were selected based on study purpose with the expectation that each participant would provide unique and rich information to the study. First, we selected Kurigram district (a northern border region of Bangladesh). Then, two upazila towns (second lowest tier of regional administration in Bangladesh) from this district, Phulbari and Kurigram sadar, were selected for urban sample. Six unions (smallest administrative classification of Bangladesh), namely Naodanga, Kashipur, Phulbari, Bara Bhita, Bhangamore and Shimulbari union under these two upazilas were selected for the rural sample. Primary data were collected from homemakers who were in the age group of 15–49 years during the period October 18, 2020 to December 10, 2020. Three expert female and three expert male interviewers collected the data by conducting a face-to-face survey. Those who fell in the age group of 15–49 years and were willing to provide information were included in the sample. Women who had mental and severe health problems, were pregnant, widowed, divorced, or separated, underwent hysterectomy, reported infertility and menopause were excluded from the sample. Consent was also obtained from parents or guardians of minors. The sample size was determined using the formula for cross-sectional study, *N* = *Z*^2^*PQ*/*D*^2^, where *N* is the required sample size, *Z* is 1.96 at 95% confidence interval (CI), *D* is the margin of error at 5% (standard deviation of 0.05), and *Q* = 1 − *P*. Since this study was the first of its type in Bangladesh, *P* = 50% was used. A minimum sample size of 423 women was obtained. The larger the target sample size, the higher the external validity and the greater the generalizability of the study [14]. Hence, this study aimed to maximize the sample size, collecting data from as many respondents as possible. The interviewers visited the assigned households and interviewed eligible respondents. If there were no eligible respondents at the household visited, the house next to it was visited. If no eligible women were found, the interview was abandoned. Out of 2665 women who were asked to participate in the interview process, 668 refused. Finally, data of 1990 individuals were included in the analysis due to some of the missing values in the dataset.

A structured questionnaire was prepared and provided to the interviewers for data collection. Before data collection, a pretest was conducted among 100 women (5% of the sample size) who were not included in the study. Based on the pretest findings, ambiguous questions were amended. Formative review for completeness and consistency of responses was being continuously made by the supervisors on a daily basis. Socio-demographic data such as age, education level, current work status, house type, family member, monthly family income, religion, family type, locality, etc. were collected by close-ended questions. Some information of respondents’ husbands, such as education level, occupation, income, were also collected.

Some close-ended (yes/no type) questions were included in the questionnaire as well to collect binominal type data such as whether they (respondents) are currently using FP, have ever used FP or oral contraceptive pills (OCP), are pregnant now, have ever conceived unexpectedly, have taken the unwanted child, have ever had miscarriage/abortion record. Basic information and information of FP use, materials, side effects, drinking water, toilet/sanitation, marriage duration, number of children, number of premature child if any, reasons for not using FP were collected with categorical outcomes questions.

### 2.2 Data analysis

IBM SPSS Statistics 25.0, Microsoft Excel 16 were mainly used to analyze the data. For the binominal outcomes, yes = 1 and no = 0 were recorded. For ordinal questions with more than two outcomes, 1, 2, 3… were recorded according to the responses. In addition, non-parametric chi-square test, binary logistic regression and simple descriptive tests were performed to analyze the data. All tests were done at the 95% confidence intervals (5% level of significance) and the two-sided significance value (*P*-value) was considered statistically significant.

### 2.3 Ethics statement

This research protocol was evaluated and approved by Research Ethical Committee (REC) of Department of Biochemistry and Food Analysis, Patuakhali Science and Technology University, Bangladesh (Approval Number: BFA: 13/09/2020:04). Each of the surveyed individuals was aware of the purpose and further maneuver of the collected data, and written agreements justifying the consent are kept.

## 3. Results

### 3.1 Socio-demographic and economic characteristics

A total of 1990 women aged between 15 and 49 years were included in the study with a response rate of (74.93%). More than three-quarters of the women were currently working (79.45%) and had 1–5 family members (80.40%) (Table 1). One-fourth of the respondents (25.93%) had an education level of bachelor’s degree or above. 74.97% (three-fourth) lived in a nuclear family, while 75.43% (three-fourth) had a BDT 15,000 or above monthly income. The majority of the respondents (90.05%) were Muslim, and about 50.20% lived in rural areas. The Chi-square test was used to examine the associations between the dependent variable–the current use of FP–and each independent variables. Out of all the examined variables, age, current work status, number of family members, locality (rural vs urban) and husbands’ education level exhibited significant associations with the current use of FP. The results of those variables are given in Table 1. Some basic information and other characteristics of the respondents and their husbands are summarized in S1 Table.

**Table 1:** Scio-demographic and socio-economic characteristics of the respondents.

Variables	Categories	Currently using family planning				Total		P-value
		Yes		No				
		n	%	n	%	n	%	
Age	15–24 Year	191	9.60	214	10.75	405	20.35	
	25–34 Year	290	14.57	526	26.43	816	41.01	<0.001*
	35–49 Year	236	11.86	533	26.78	769	38.64	
Educational level	No school attended	1	0.05	9	0.45	10	0.50	
	Between 1 and 5	48	2.41	122	6.13	170	8.54	
	between 6 and 9	147	7.39	283	14.22	430	21.61	
	SSC completed	157	7.89	271	13.62	428	21.51	0.057
	Higher Secondary	161	8.09	275	13.82	436	21.91	
	Bachelor and above	203	10.20	313	15.73	516	25.93	
Current work status	Not Working	588	29.55	993	49.90	1581	79.45	0.034*
	Working	129	6.48	280	14.07	409	20.55	** **
Family member	1–5 Members	598	30.05	1002	50.35	1600	80.40	0.011*
	Above 5	119	5.98	271	13.62	390	19.60	
Monthly family income	5000–10000	15	0.75	25	1.26	40	2.01	
	10000–15000	146	7.34	303	15.23	449	22.56	0.211
	Above 15000	556	27.94	945	47.49	1501	75.43	
Socioeconomic status	Upper	556	27.94	945	47.49	1501	75.43	
	Middle	118	5.93	221	11.11	339	17.04	0.110
	Lower	43	2.16	107	5.38	150	7.54	
Religion	Islam	639	32.11	1153	57.94	1792	90.05	0.299
	Hindu	78	3.92	120	6.03	198	9.95	
Family type	Nuclear	521	26.18	971	48.79	1492	74.97	0.074
	Joint	196	9.85	302	15.18	498	25.03	
Locality	Rural	295	14.82	704	35.38	999	50.20	<0.001*
	Urban	422	21.21	569	28.59	991	49.80	
Husbands’ education level	No school attended	5	0.25	15	0.75	20	1.01	
	Between 1 and 5	19	0.95	41	2.06	60	3.02	
	between 6 and 9	125	6.28	274	13.77	399	20.05	
	SSC completed	69	3.47	151	7.59	220	11.06	0.003*
	Higher Secondary	133	6.68	264	13.27	397	19.95	
	Bachelor and above	366	18.39	528	26.53	894	44.92	
Husbands’ occupation	Govt. service	117	5.88	202	10.15	319	16.03	
	Private or other service	194	9.75	274	13.77	468	23.52	
	Farmer	9	0.45	21	1.06	30	1.51	
	Business	190	9.55	334	16.78	524	26.33	
	Day Labor	49	2.46	81	4.07	130	6.53	0.057
	Fishermen	3	0.15	7	0.35	10	0.50	
	Wood Cutter	6	0.30	14	0.70	20	1.01	
	Other	149	7.49	340	17.09	489	24.57	

Note: “n” represents the number of respondents, “*” indicates significance at the 5% level.

### 3.2 Sources of information regarding FP methods during COVID-19 pandemic

**Fig 1:**
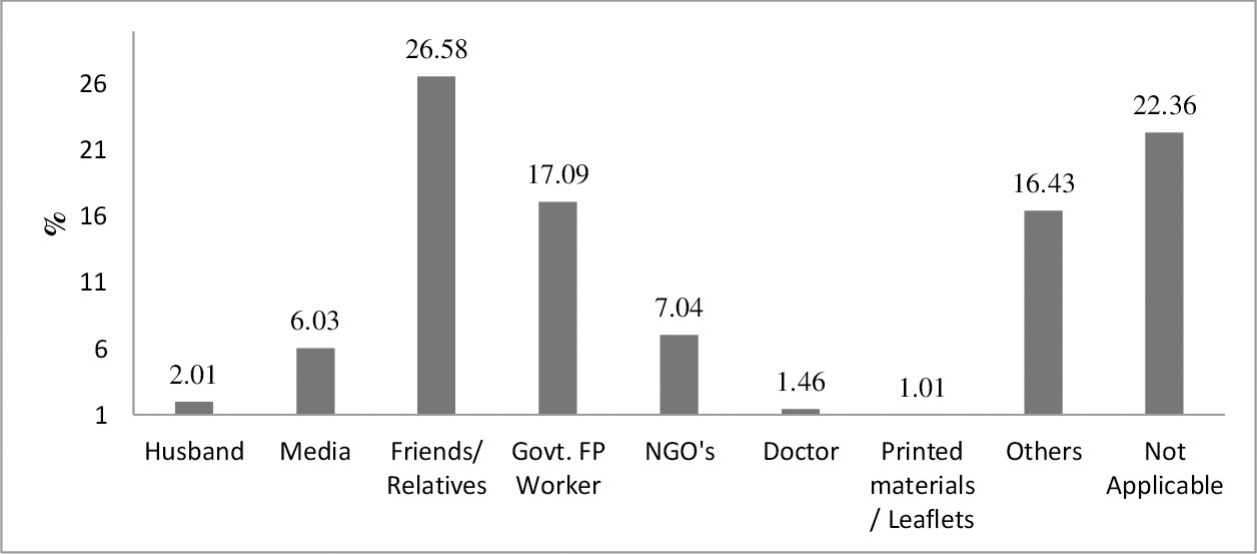
Sources of information regarding family planning methods during COVID-19 pandemic.

The primary sources of information about FP were friends/relatives (26.58%), government FP workers (17.09%), and NGOs (7.04%) (Fig 1). Media, husbands, doctors, newspapers, and leaflets had limited contribution to disseminating FP information. Among the respondents, 16.43% indicated other sources of information, while 22.36% answered the question “does not apply.”

### 3.3 Prevalence of utilization of FP methods during COVID-19 pandemic

**Fig 2:**
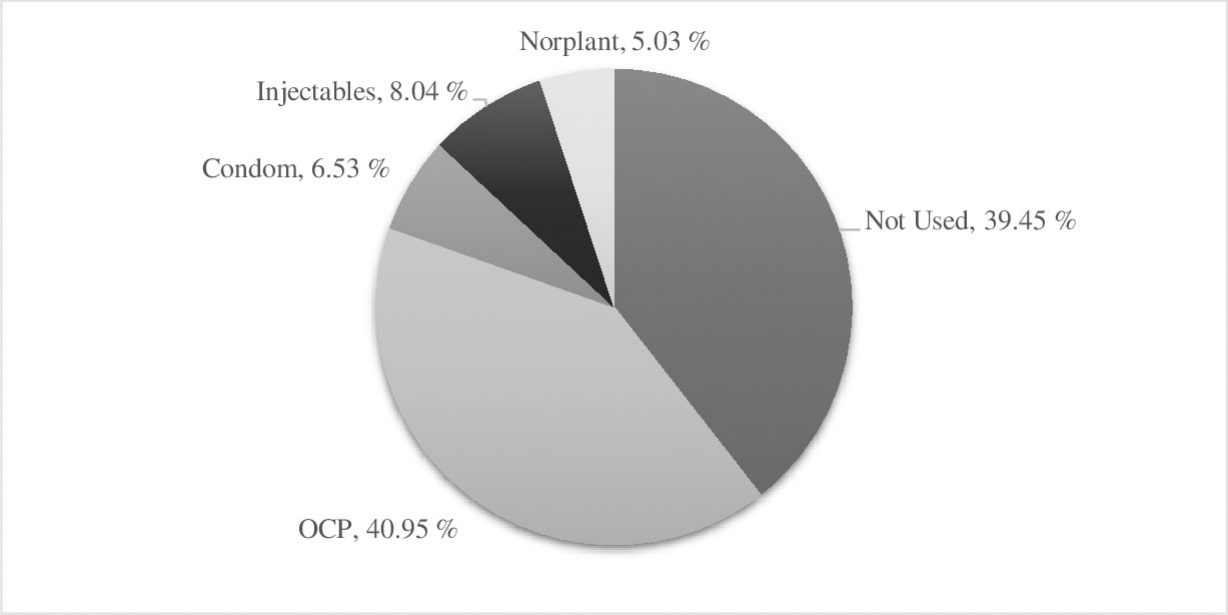
Preferred types of family planning methods (modern) during COVID-19 pandemic.

**Fig 3:**
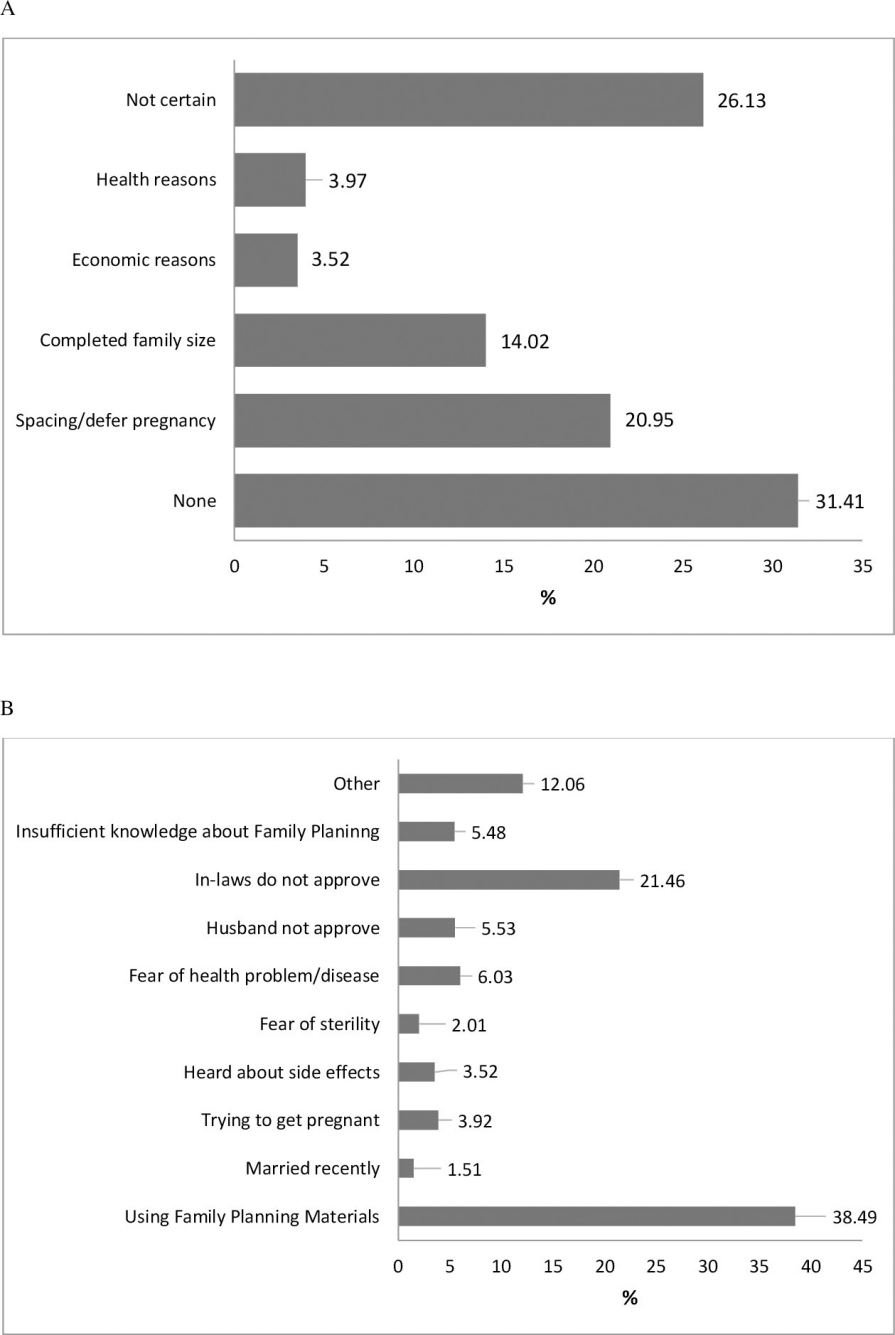
Reasons for using (3A) and non-using (3B) family planning methods during COVID-19 pandemic.

In this study, the level of utilization of FP methods was 36.03% among the respondents. Of them, about 40.95% were using OCP, 8.04% injectable, 6.53% condom, and 5.03% Norplant (Fig 2). 44.67% and 1.01% used to have a safe period and withdrawal from the traditional method, respectively (S1 Fig). Additionally, 20.92% indicated that FP was due to spacing/defer pregnancy, and another 14.02% indicated that it was for already having completed family size (Fig 3A). Reasons for not using contraceptives were investigated in an open-ended question for women not using them (Fig 3B). We found that “in-laws ‘disapproval” was the first and “other” was the second most reported reasons for not using FP (21.46% and 12.06%, respectively). The most frequently reported side effects of FP use were weight gain (17.44%) and headache (11.57%) (S2 Fig).

### 3.4 Factors associated with the use of FP methods during COVID-19 pandemic

The adjusted estimated effects of the factors associated with FP use are presented in Table 2. The results show that age, education level of the respondents, working status of the household head, locality (rural vs urban), reading newspaper, FP workers’ advice, currently using OCP, ever used OCP, husbands’ supports towards OCP, duration of marriage, ever pregnant, number of children, and dead child show significant association with current FP use.

**Table 2:** Regression model for the factors affecting currently used FP during COVID 19 pandemic.

Predictors	Categories	P-value	[COR, 95% CI (LL-UL)]	P-value	[AOR, 95% CI (LL-UL)]
Age	15–24 Year	<0.001	2.02 [1.57–2.58]	**0.002** *	1.85 [1.26–2.73]
	25–34 Year	0.041	1.25 [1.01–1.54]	0.985	1.00 [0.74–1.37]
	35–49 Year	Ref.			
Education level	Below 5	0.004	0.58 [0.40–0.84]	0.336	1.46 [0.68–3.16]
	between 6 and 9	0.102	0.80 [0.61–1.05]	0.070	1.63 [0.96–2.75]
	SSC completed	0.403	0.89 [0.69–1.16]	**0.022** *	1.65 [1.08–2.53]
	Higher Secondary	0.445	0.90 [0.69–1.17]	0.166	1.29 [0.90–1.85]
	Bachelor and above	Ref.			
Husbands’ education level	Below 5	0.058	0.62 [0.38–1.02]	0.315	1.58 [0.65–3.82]
	between 6 and 9	0.001	0.66 [0.51–0.85]	0.790	1.07 [0.64–1.81]
	SSC completed	0.009	0.66 [0.48–0.90]	0.388	1.29 [0.72–2.29]
	Higher Secondary	0.011	0.73 [0.57–0.93]	0.354	0.84 [0.58–1.21]
	Bachelor and above	Ref.			
Family member	1–5 Members	0.012	1.36 [1.07–1.72]	0.389	1.17 [0.82–1.68]
	Above 5	Ref.			
Work status of household head	Govt. service	0.067	1.32 [0.98–1.78]	0.100	1.44 [0.93–2.21]
	Private or other service	<0.001	1.62 [1.24–2.11]	**0.045** *	1.50 [1.01–2.22]
	Farmer/ Fishermen/Wood cutter	0.940	0.98 [0.54–1.76]	0.487	1.31 [0.61–2.85]
	Business	0.051	1.30 [1.00–1.69]	**0.050** *	1.44 [1.00–2.06]
	Day Labor	0.117	1.38 [0.92–2.07]	0.078	1.75 [0.94–3.25]
	Other	Ref.			
Monthly family income	5000–10000	0.953	1.02 [0.53–1.95]	0.369	0.62 [0.22–1.77]
	10000–15000	0.080	0.82 [0.65–1.02]	0.545	0.89 [0.60–1.31]
	Above 15000	Ref.			
Religion	Islam	0.299	0.85 [0.63–1.15]	0.718	1.08 [0.71–1.63]
	Hindu	Ref.			
Family type	Nuclear	0.074	0.83 [0.67–1.02]	0.333	0.85 [0.62–1.18]
	Joint	Ref.			
Locality	Rural	<0.001	0.57 [0.47–0.68]	**<0.001** *	0.35 [0.25–0.49]
	Urban	Ref.			
Reading newspaper at least	Don’t Read	<0.001	0.64 [0.52–0.78]	**<0.001** *	0.33 [0.25–0.45]
	Less than Once a week	0.071	0.65 [0.40–1.04]	**0.004** *	0.37 [0.18–0.73]
	Above Once a week	Ref.			
Ever pregnant	Yes	<0.001	0.41 [0.34–0.51]	**<0.001** *	0.23 [0.17–0.32]
	No	Ref.			
Currently using OCP	Yes	<0.001	8.79 [6.96–11.1]	**<0.001** *	30.79 [19.1–49.4]
	No	Ref.			
Family planning worker advice for family planning	Yes	<0.001	1.48 [1.20–1.83]	**<0.001** *	2.68 [1.99–3.62]
	No	Ref.			
Dead child	None	0.003	1.91 [1.26–2.91]	**0.001** *	2.54 [1.47–4.39]
	One	Ref.			
Are you pregnant now?	Yes	0.807	0.96 [0.70–1.32]	0.061	1.49 [0.98–2.26]
	No	Ref.			
For abortion have any physical/mental problems	Yes	0.031	1.64 [1.05–2.57]	0.238	0.68 [0.35–1.30]
	No	Ref.			
Duration of marriage (year)	Below 10	<0.001	10.80 [7.6–15.2]	**<0.001** *	6.44 [4.08–10.17]
	10–20	<0.001	6.20 [4.25–9.04]	**<0.001** *	4.04 [2.57–6.36]
	Above 20	Ref.			
Ever use OCP	Yes	<0.001	2.31 [1.91–2.78]	**<0.001** *	0.12 [0.07–0.20]
	No	Ref.			
Using OCP husband’s attitude is supportive	Yes	<0.001	2.89 [2.39–3.50]	**<0.001** *	1.95 [1.30–2.94]
	No	Ref.			
No. of children	None	<0.001	4.67 [3.28–6.66]	0.072	0.59 [0.33–1.05]
	Below Two	<0.001	4.62 [3.35–6.35]	**0.007** *	1.86 [1.18–2.94]
	Three or Plus	Ref.			

Note

“*” indicates significance at the 5% level. AOR stands for adjusted odds ratio, and COR crude odds ratio. CI is confidence interval. Here, dependent variable is “currently using FP”, and reference is “not using FP. (LL-UL) states LL: Lower limit, and UL: Upper limit.

Women’s age, as expected, plays a vital role in the use of FP. The 20–24 years aged women had a 1.85 times higher chance of using contraception than the 35–49 years aged group (AOR: 1.85, 95% CI: 1.26–2.73). The odds of women with secondary level education were 1.65 times higher compared with women having a bachelor’s degree or above (AOR: 1.65; 95% CI: 1.08–2.53). If the household head worked in private sectors or did business, there was a 1.50 times higher chance of FP use than the household head working in other services (AOR: 1.50; 95% CI: 1.01–2.22). Similarly, the odds were almost 1.44 times higher for household head doing business than household heads working in other services (AOR: 1.44; 95% CI: 1.00–2.06). Place of living also had a significant influence on FP use. For instance, it was found that a woman living in rural areas had approximately 65% less chance of FP use compared to their counterparts living in urban areas. A woman who read a newspaper once in a week and those who did not read had 63% and 67% lower chance of FP use respectively than a woman reading a newspaper more than once in a week (AOR: 0.37, 95% CI: 0.18–0.73 and AOR: 0.33, 95% CI: 0.25–0.45). Women following FP workers’ advice for FP had a 2.68 times higher chance of FP use than women not following FP workers’ advice (AOR: 2.68, 95% CI: 1.99–3.62).

For women currently using OCP, the chance of using FP was approximately 30.79 times higher than those not currently using OCP. We also found that a woman who had experience of ever using OCP had an 88% lower chance of FP use than a woman who had no experience of OCP use (AOR: 0.12, 95% CI: 0.07–0.20). As deemed, the woman using OCP and receiving supportive attitude from husband, the chance of FP use was 1.95 times higher compared with those not receiving supportive attitude from husband (AOR: 1.95, 95% CI: 1.30–2.94).

Moreover, women having a marriage duration of 10–20 years had about four times higher chance of FP use than women having more than 20 years of married life (AOR: 4.04, 95% CI: 2.57–5.36), whereas the chance is 6.44 times higher for the woman whose married life was less than 10 years (AOR: 6.44, 95% CI: 4.08–10.17). Furthermore, if women were ever pregnant, there was a 77% lower chance of FP use than those who were not pregnant (AOR: 0.23, 95% CI: 0.17–0.32). Moreover, if the number of women’s children was lower than two, the chance of FP use was close to two times higher than the number of those having three or more children (AOR: 1.86, 95% CI: 1.18–2.94). Lastly, women whose child died before had more than twice the chance of FP use than women whose child never died (AOR: 2.54, 95% CI: 1.47–4.39).

## 4. Discussion

This community based cross-sectional study was conducted to assess the utilization of FP methods and associated factors among women who were in the age group of 15–49 years. Consistent with other studies, the result of this study found that friends/relatives and government FP workers were the most trusted sources of FP-related information [15–17]. Before the pandemic, the prevalence of FP usage in Bangladesh was 62% which is very close to the world contraceptive use prevalence (64%) [18]. However, this rate is higher than in other South Asian countries. For example, FP use rate is 58% in India, 50% in Nepal, 35% in Pakistan, and 23% in Afghanistan [19–23]. This rate is low compared to Thailand (79%) and Singapore (74%) [24]. The COVID-19 pandemic has lowered the FP use rate in the study areas by 23%, giving a current rate of 36.03%.

The use of contraceptives is highly related to family size and spacing between births [25–28]. This study found that complete desired family size and spacing between births are the main reasons for using FP methods during the COVID-19 pandemic. According to a recent study, fear of adverse effects is the most common reason for not using FP, followed by religious concerns and disapproval from husbands or families [29]. However, this study found in-laws’ disapproval as a significant reason for not using FP methods. The use of the FP method also varies across women of different ages. With increasing age, the need and rate of women’s FP use decreases [30, 31]. Our findings also revealed that women aged 20–24 years have an increased chance of using FP than women aged 35–49 years.

Some studies have demonstrated an association between educational attainment and contraceptive use [32]. It is possible that less educated women are less likely to have higher job ambitions and a poor awareness of health, resulting in a lack of incentive to utilize FP. However, education was also shown to have no effect on FP use in some other studies. For example, studies from 9,134 Australian women [33], and Bangladesh [18] showed no associations between overall FP use and education. This study revealed that current FP use was higher among women with secondary level education than women with a bachelor’s degree or above. This scenario is unique and remains *unclear*, that *needs further exploration*.

Our results suggest that if the household heads were working in private sectors or doing business, there was a higher chance of FP use among women in those households with their counterparts whose household heads were working in other services. The use of FP varies geographically, either with administrative or domain levels [34, 35]. These studies consistently reported that urban areas had a higher prevalence of FP use than rural areas due to the differences in socioeconomic conditions, the visit of FP workers, the level of women’s autonomy, and other community-level characteristics. The current study found similar results that show FP use varies with places of residence, with rural areas having higher prevalence of FP use than urban areas.

Access to media, for example, frequency of reading newspapers, is hypothesized to influence FP use in a previous study conducted in Kenya [36]. In line with this argument, reading newspapers was an important predictor of current FP use in this study. FP workers’ visit is also assumed to influence the FP use rate as suggested by some previous studies [37–39]. Previous studies from Bangladesh found that discontinuation of services from FP workers were associated with higher discontinuation of contraceptive use [39, 40]. Following prior studies, this study also found higher use of FP among women frequently visited by FP workers.

Many factors would influence the impact of the COVID-19 pandemic on meeting the demand for FP. One such factor is the type of contraceptive methods used by women across countries. The findings of this study revealed that 24.42% of the respondents are currently using OCP, which is much lower than 61.7% reported by a prior study conducted in Bangladesh before the pandemic. This rapid decline in OCP may potentially indicate the possible discontinuity of FP workers’ visit, the periodicity of renewal, the susceptibility to stock-outs, and global supply chains disruptions amid COVID-19 pandemic [18]. Inconsistent with earlier studies, this study found that women who ever used OCP were less likely to use FP compared to women who never used OCP [41]. This can be because previous users of OCP were more familiar with its detrimental effects. There are some sub-groups of women in Bangladesh whose husbands’ support of FP use is still a significant barrier. The supportive attitude of husbands is the most significant predictor of current FP use in Bangladesh [42]. In line with previous studies, the current study found that if husbands were positive towards OCP use, odds of current FP use would increase significantly.

Our current research suggests a higher rate of contraceptive use over the first 20 years of marriage, followed by a decline. This accords with the study by Shree et al. (2017) that found higher contraceptive use among women with 10–20 years of marriage duration, and thereafter there was a fall [43]. Furthermore, women who reported having pregnancy before were less likely to use FP than women who never had a pregnancy [44]. Our results are in accord with this argument. In addition, significant variations in contraceptive use were found across women with different number of children in some previous studies [18, 35]. We found that women whose child died before and the living was lower than two had more chance of FP use.

Our study has several limitations. The main limitation is that there were not enough studies conducted during the pandemic to compare the results. Moreover, it is a cross-sectional study, thus depicting a picture of the community response at a point of time. Moreover, there might be a bias from the respondents’ side in answering questions. For example, some women may have reported the modern method, while they actually used the traditional method and vice-versa. Another difficulty was that many women felt shy and hesitate to provide their confidential information. Some of them directly refused to provide answers. Furthermore, although we used a demographically representative sample of the Bangladeshi population, we could not be sure how representative the survey respondents were in terms of the mass population. There could also be many other parameters that influenced FP use, which was not included into our study.

## 5. Conclusions

This study examined the prevalence and factors associated with FP use among women between 15–49 years of age. FP use among women was found to be decreased in Bangladesh amid the COVID-19 pandemic. Age, education level of the respondents, working status of the household heads, locality, reading the newspaper, FP workers’ advice, currently using OCP, ever used OCP, husbands’ supportive attitude towards OCP use, duration of the marriage, ever pregnant, number of the children, and dead child were found to be important predictors of utilization of FP in this study. Therefore, communication between husbands and wives on FP matters is an essential intermediate step along with other ways of promoting the sustained use of FP methods. Based on local contexts, pragmatic approaches to FP programs are needed to increase the FP use rate in the places that are lagging behind. In addition, building awareness of the benefits of having a smaller family would positively impact increasing FP use. The FP workers and stakeholders should motivate women with unmet needs to adopt permanent methods of FP by explaining to them the related benefits of using those methods. The above guidance provides a foundation for continuing safe contraceptive service provision that countries may adapt based on the local contexts taking into account local policies and stages of the epidemic.

## Supporting information

S1 File English version of the questionnaire. (DOCX)

S2 File Bengali version of the questionnaire. (PDF)

S1 Table Basic information of the respondents. (DOCX)

S1 Fig Preferred types of family planning method (traditional) during COVID-19 pandemic. (TIF)

S2 Fig Side effects of family planning methods during COVID-19 pandemic. (TIF)
