# Supplementary material for: Prevalence and factors associated with family planning during COVID-19 pandemic in Bangladesh: A cross-sectional study
Source: PLoS One. 2021 Sep 21;16(9):e0257634. doi: 10.1371/journal.pone.0257634 (PMC8454962; doi:10.1371/journal.pone.0257634)
Supplement: S1 File — (DOCX) [file pone.0257634.s001.docx]

**Title: Prevalence and factors associated with family planning during COVID-19 pandemic in Bangladesh: a cross-sectional study**

Date………………

Name of surveyor……………………………………………..

Code of participant: ……………..

**PART A. Socio-demographic & Socio-economic information**

|  | Age (years): | 1. 15 - 24 b. 25 - 34 c. 35 - 49 |
| --- | --- | --- |
|  | Can you read Bangla? | 1. Yes b. No |
|  | Can you write Bangla? | 1. Yes b. No |
|  | What is your education level? | a. No school attended b. between 1 and 5 c. Between 6 and 9 d. SSC completed e. Higher Secondary f. Bachelor and above |
|  | What is your current working status? | a. Not working b. Working |
|  | What is your husband’s education level? | a. No school attended b. between 1 and 5  c. Between 6 and 9 d. SSC completed e. Higher Secondary f. Bachelor and above |
|  | How many family members do you have? | 1. 1 - 5 Members b. Above 5 |
|  | What is your husbands’ occupation? | 1. Govt. service b. Private or other service 2. Farmer c. Business d. Day labor 3. Fishing e. Wood cutter f. Others |
|  | What is your monthly family incomes (BDT) | 1. 5000-10000 b. 10000-15000   c. Above 15000 |
|  | Socioeconomic status (based on family income) | 1. Upper b. Middle c. Lower |
|  | Religion | 1. Islam b. Hindu |
|  | Type of family | 1. Nuclear b. Joint |
|  | Locality | 1. Rural b. Urban |
|  | House | 1. Personal b. Rent c. Other |
|  | Housing Construction | 1. Pucca b. Semi Pucca c. Katcha |
|  | Drinking water sources | 1. Tube-Well b. Pipe Water c. Other |
|  | Domestic use water sources | 1. Pond/tank/lake b. River/stream   c. Tube well d. Piped water  e. Other sources |
|  | Toilet/Sanitation | 1. Septic tank/Modern latrine 2. Water Sealed/Slab latrine c. Pit latrine 3. Open latrine e. Hanging latrine |
|  | Get daily newspaper | 1. Yes b. No |
|  | Reading newspaper at least | 1. Don’t read b. Less than once a week   c. Above once a week |
|  | Watching television | 1. Yes b. No |

**PART B. Women’s Personal Information**

|  | Duration of marriage (years) | 1. Below 10 b. 10 – 20 c. Above 20 |
| --- | --- | --- |
|  | Ever pregnant | 1. Yes b. No |
|  | Ever used family planning | 1. Yes b. No |
|  | Currently using family planning | 1. Yes b. No |
|  | Preferred types of FP method (Modern) | 1. Oral pill b. Condom c. Injectable   d. Norplant e. Not used |
|  | Preferred types of FP method (Traditional) | 1. Safe period b. Withdrawal c. Not used |
|  | Ever used oral contraceptive pill | 1. Yes b. No |
|  | Currently using oral contraceptive pill | 1. Yes b. No |
|  | What is the sources from which you informed about FP? | 1. Husband b. Media c. Friends and relatives   d. GOB FP worker e. NGO health worker  f. Doctor g. Printed material/ leaﬂet  h. Others i. Not applicable |
|  | Did any FP worker come your home for advising FP use? | 1. Yes b. No |
|  | Are you Involved in any NGO programs (credit, income generating activities, other)? | 1. Yes b. No |
|  | For using OCP your husband’s attitude is Supportive? | 1. Yes b. No |
|  | No. of children | 1. None b. One c. Two d. Three. 2. Four/more than four |
|  | How many of your children are wanted? | 1. None b. One c. Two |
|  | How many of your children are unwanted? | 1. None b. One c. Two |
|  | How many of your children are mistimed? | 1. None b. One c. Two |
|  | No. of living children | 1. None b. 0-1 c. 2-3 d. 4+ |
|  | Did you have a dead child? | 1. None b. One |
|  | sex composition of living children: | 1. No child b. Only daughter   c. Only son d. Both |
|  | Which reasons for utilizing various family planning methods? | 1. Spacing/defer pregnancy   b. Completed family size c. Economic reasons  d. Health reasons e. Not certain f. None |
|  | Are you pregnant now? | 1. Yes b. No |
|  | Have you ever conceived unexpectedly? | 1. Yes b. No |
|  | Did you take the unwanted child? | 1. Yes b. No |
|  | Whether or not miscarriage/abortion it? | 1. Yes b. No |
|  | For abortion the child do you have any physical/mental problems? | 1. Yes b. No |
|  | Reasons for non-use of FP | 1. Married recently b. Trying to get pregnant   c. Heard about side-effects d. Fear of sterility   1. Fear of health problems/disease 2. Husband not approve 3. In-laws do not approve 4. Insufﬁcient knowledge about FP 5. Other |
