# Supplementary material for: Prevalence and factors associated with family planning during COVID-19 pandemic in Bangladesh: A cross-sectional study
Source: PLoS One. 2021 Sep 21;16(9):e0257634. doi: 10.1371/journal.pone.0257634 (PMC8454962; doi:10.1371/journal.pone.0257634)
Supplement: S2 File — (PDF) [file pone.0257634.s002.pdf]

বাংলাদেশে কোভিড -১৯ মহামারী চলাকালীন পরিবার পরিকল্পনার সাথে জড়িত বিস্তার এবং কারণগুলি: একটি ক্রস-বিভাগীয় গবেষণা

তারিখ :

জরিপকারীর নাম :

অংশগ্রহণকারীর কোড:

ক অংশ: সামাজিক-জনসংখ্যাতাত্ত্বিক এবং আর্থ-সামাজিক তথ্য:

১. বয়স (বছর):	ক) ১৪-২৪ খ) ২৫-৩৪ গ) ৩৫-৪৯
২. আপনি কি বাংলা পড়তে পারেন ?	ক) হ্যাঁ খ) না
৩. আপনি কি বাংলা লিখতে পারেন ?	ক) হ্যাঁ খ) না
৪. আপনার শিক্ষাগত যোগ্যতা কোনটি ?	ক) কখনো বিদ্যালয়ে যাওয়া হয় নাই খ) প্রথম এবং পঞ্চম শ্রেণী এর মধ্যে গ) ষষ্ঠ এবং নবম শ্রণী এর মধ্যে ঘ) মাধ্যমিক<br>ঙ) উচ্চমাধ্যমিক চ) স্নাতক এবং এর উপরে
৫. বর্তমান আপনি কি কোন কাজ করেন ?	ক) কোন কাজ করি না খ) কাজ করি
৬. আপনার স্বামীর শিক্ষাগত যোগ্যতা কোনটি ?	ক) কখনো বিদ্যালয়ে যাওয়া হয় নাই খ) প্রথম এবং পঞ্চম শ্রেণী এর মধ্যে গ) ষষ্ঠ এবং নবম শ্রণী এর মধ্যে ঘ) মাধ্যমিক<br>ঙ) উচ্চমাধ্যমিক চ) স্নাতক এবং এর উপরে
৭. আপনার পরিবারের সদস্য সংখ্যা কতজন ?	ক) ১-৫ জন খ) ৫ এর বেশী
৮. আপনার স্বামীর পেশা কোনটি ?	ক) সরকারী চাকুরি খ) বেসরকারী চাকুরি গ) কৃষক ঘ) ব্যবসায়ী ঙ) দিনমুজুর চ) জেলে ছ) কাঠুরিয়া জ) অন্যান্য
৯. আপনার পরিবারের মাসিক আয় কত টাকা?	ক) ৫০০০-১০০০০ টাকা খ) ১০০০০-১৫০০০ টাকা<br>গ) ১৫০০০ টাকা এর বেশী
১০. অর্থসামাজিক অবস্থা :	ক) উচ্চবিত্ত খ) মধ্যবিত্ত গ) নিম্নবিত্ত
১১. ধর্ম :	ক) ইসলাম খ) হিন্দু
১২. পরিবারের ধরন:	ক) একক পরিবার খ) যৌথ পরিবার
১৩. এলাকা:	ক) শহর খ) গ্রাম
১৪. বসতবাড়ি:	ক) নিজস্ব খ) ভাড়া গ) অন্যান্য
১৫. বসতবাড়ি কাটামো:	ক) পাকা খ) অর্ধ পাকা গ) কাচা
১৬. খাওয়ার পানির উৎস:	ক) টিউবওয়েল খ) পাইপ লাইন এর পানি গ) অন্যান্য
১৭. গৃহস্থলির কাজের পানির উৎস:	ক) পুকুর খ) নদী গ) টিউবওয়েল<br>ঘ) পাইপ লাইন এর পানি ঙ) অন্যান্য উৎস
১৮. টয়লেট/স্যানিটেশন:	ক) আধুনিক টয়লেট খ) স্লাব টয়লেট<br>গ) ছোট গর্ত করা টয়লেট ঘ) খোলা টয়লেট<br>ঙ) ঝুলানো টয়লেট
১৯. দৈনিক সংবাদপত্র পান:	ক) হ্যাঁ খ) না
২০. সপ্তাহে অন্তত সংবাদপত্র পড়া:	ক) পড়েন না খ) সপ্তাহে ১ দিন গ) সপ্তাহে ১ দিন এর বেশী
২১. টেলিভিশন দেখেন:	ক) হ্যাঁ খ) না

খ অংশ: মহিলাদের ব্যক্তিগত তথ্য

১. বিয়ের সময়কাল (বছর) :	ক) ১০ বছরের কম খ) ১০-২০ বছর গ) ২০ বছর এর বেশী
২. কখনো গর্ভবতী হয়েছেন:	ক) হ্যাঁ খ) না
৩. কখনো পরিবার পরিকল্পনা ব্যবহার করেছেন:	ক) হ্যাঁ খ) না
৪. বর্তমান পরিবার পরিকল্পনা ব্যবহার করছেন:	ক) হ্যাঁ খ) না
৫. পছন্দের পরিবার পরিকল্পনা পদ্ধতি (আধুনিক):	ক) গর্ভনিরোধক পিল খ) কন্ডম গ) ইনজেকশন ঘ) নরপ্লান্ট ঙ)ব্যবহার করি না
৬. পছন্দের পরিবার পরিকল্পনা পদ্ধতি (গতানুগতিক):	ক) নিরাপদ সময়কাল খ) সরিয়ে নেয়া গ) ব্যবহার করি না
৭. কখনো গর্ভনিরোধক পিল ব্যবহার করছেন:	ক) হ্যাঁ খ) না
৮. বর্তমান কোন গর্ভনিরোধক পিল ব্যবহার করেন:	ক) হ্যাঁ খ) না
৯. আপনি কোন মাধ্যমে পরিবার পরিকল্পনা এর ব্যাপারে জেনেছেন ?	ক) স্বামী খ) প্রচার মাধ্যম গ) বন্ধু এবং আত্মীয়স্বজন ঘ) সরকারী পরিবার পরিকল্পনা কর্মী ঙ) এনজিও স্বাস্থ্য কর্মী চ) ডাক্তার ছ) সংবাদপত্র জ) কোন কিছুই না
১০. পরিবার পরিকল্পনা এর ব্যাপারে উপদেশ দিতে কোন কর্মী আপনার বাড়িতে এসেছিল কি না ?	ক) হ্যাঁ খ) না
১১. আপনি কি কোন এনজিও এর প্রোগ্রামের সাথে জড়িত (ক্রেডিট, আয় উৎপাদন কার্যক্রম, অন্যান্য) ?	ক) হ্যাঁ খ) না
১২. গর্ভনিরোধক পিল ব্যবহারে আপনার স্বামীর মনোভাব সহায়ক ছিল কি না ?	ক) হ্যাঁ খ) না
১৩. সন্তান এর সংখ্যা:	ক) ১ জনও না খ)১ জন গ) ২ জন ঘ)৩ জন ঙ) ৪ জন অথবা এর বেশী
১৪. আপনার সন্তানদের মধ্যে কতজন কাঙ্ক্ষিত ছিল?	ক) ১ জনও না খ)১ জন গ) ২ জন
১৫. আপনার সন্তানদের মধ্যে কতজন অনাকাঙ্ক্ষিত ছিল ?	ক) ১ জনও না খ)১ জন গ) ২ জন
১৬. আপনার সন্তানদের মধ্যে কতজন অনুপযুক্ত মুহূর্তে এসেছিল?	ক) ১ জনও না খ)১ জন গ) ২ জন
১৭. জীবীত সন্তানদের সংখ্যা:	ক) নেই খ) ০-১জন গ) ২-৩ জন ঘ) ৪ জন এর বেশী
১৮. আপনার কি মৃত সন্তান ছিল ?	ক)১ জনও না খ) ১ জন
১৯. জীবিত সন্তানদের লিঙ্গ গঠন:	ক) কোন সন্তান নেই খ) শধু মেয়ে সন্তান গ) শধু ছেলে সন্তান ঘ) উভয়ই
২০.বিভিন্ন ধরনের পরিবার পরিকল্পনা পদ্ধতি ব্যবহার করার কারণ কোনটি ?	ক) গর্ভাবস্থা ফাঁকা/স্থগিত খ)পরিবারের আকার ঠিক রাখার জন্য গ)অর্থনৈতিক অবস্থা ঘ) স্বাস্থ্যগত কারনে ঙ) নিশ্চিত কারণ নেই চ) এর কোনটাই না
২১. আপনি কি বর্তমানে গর্ভবতী ?	ক) হ্যাঁ খ) না
২২. আপনি কি কখনো অপ্রত্যাশিতভাবে গর্ভধারণ	ক) হ্যাঁ খ) না

করেছেন ?	
২৩. আপনি কি অপ্রত্যাশিত শিশু সন্তানকে নিয়েছিলেন?	ক) হ্যাঁ খ) না
২৪. কখনো এই শিশুকে গর্ভপাত করেছিলেন কি না ?	ক) হ্যাঁ খ) না
২৫. গর্ভপাতের জন্য আপনার কি কোন শারীরিক অথবা মানসিক সমস্যা হয়েছিল কি না ?	ক) হ্যাঁ খ) না
২৬.পরিবার পরিকল্পনা গ্রহণ না করার কারণ গুলো কিঃ	ক) সদ্য বিবাহিত খ) গর্ভধারণ করার চেষ্টা করতেছি গ) পার্শ্বপ্রতিক্রিয়া এর কারনে ঘ) বন্ধ্যাত্বের ভয় ঙ) স্বাস্থ্যঝুকি অথবা রোগের ভয় চ) স্বামী সম্মতি দেয় না ছ) শ্বশুরবাড়ির লোকেরা অনুমোদন দেয় না জ) পরিবার পরিকল্পনা এর ব্যাপারে অপর্যাপ্ত ধারনা ঝ) অন্যান্য
