# Supplementary material for: Prevalence and factors associated with family planning during COVID-19 pandemic in Bangladesh: A cross-sectional study
Source: PLoS One. 2021 Sep 21;16(9):e0257634. doi: 10.1371/journal.pone.0257634 (PMC8454962; doi:10.1371/journal.pone.0257634)
Supplement: S1 Table — (DOCX) [file pone.0257634.s003.docx]

**S1 Table:** Basic information of the respondents

| Variables | Categories | Currently Using Family Planning  Yes No | | | | Total | | P Value |
| --- | --- | --- | --- | --- | --- | --- | --- | --- |
|  |  | n | % | n | % | N | % |  |
| Can read Bangla | Yes | 709 | 35.63 | 1241 | 62.36 | 1950 | 97.99 | **0.033*** |
|  | No | 8 | 0.40 | 32 | 1.61 | 40 | 2.01 |  |
| Can write Bangla | Yes | 692 | 34.77 | 1188 | 59.70 | 1880 | 94.47 | **0.003*** |
|  | No | 25 | 1.26 | 85 | 4.27 | 110 | 5.53 |  |
|  | Personal | 377 | 18.94 | 689 | 34.62 | 1066 | 53.57 |  |
| House | Rent | 322 | 16.18 | 553 | 27.79 | 875 | 43.97 | 0.803 |
|  | Other | 18 | 0.90 | 31 | 1.56 | 49 | 2.46 |  |
| Housing Construction | Pucca | 384 | 19.30 | 519 | 26.08 | 903 | 45.38 |  |
|  | Semi Pucca | 214 | 10.75 | 514 | 25.83 | 728 | 36.58 | **<0.001*** |
|  | Katcha | 119 | 5.98 | 240 | 12.06 | 359 | 18.04 |  |
|  | Tube-Well | 583 | 29.30 | 1072 | 53.87 | 1655 | 83.17 |  |
| Drinking water sources | Pipe Water | 104 | 5.23 | 133 | 6.68 | 237 | 11.91 | **0.018*** |
|  | Other | 30 | 1.51 | 68 | 3.42 | 98 | 4.92 |  |
|  | Pond/Tank/Lake | 174 | 8.74 | 265 | 13.32 | 439 | 22.06 |  |
|  | River/ Stream | 9 | 0.45 | 21 | 1.06 | 30 | 1.51 |  |
| Domestic use water sources | Tube-Well | 136 | 6.83 | 334 | 16.78 | 470 | 23.62 | **<0.001*** |
|  | Pipe Water | 354 | 17.79 | 627 | 31.51 | 981 | 49.30 |  |
|  | Other | 44 | 2.21 | 26 | 1.31 | 70 | 3.52 |  |
|  | Septic Tank / Modern Latrine | 516 | 25.93 | 836 | 42.01 | 1352 | 67.94 |  |
| Toilet/sanitation | Water Sealed/ Slab Latrine | 174 | 8.74 | 374 | 18.79 | 548 | 27.54 | **0.015*** |
|  | Pit / Open/ Hanging Latrine | 27 | 1.36 | 63 | 3.17 | 90 | 4.52 |  |
| Have Television | Yes | 598 | 30.05 | 967 | 48.59 | 1565 | 78.64 | **<0.001*** |
|  | No | 119 | 5.98 | 306 | 15.38 | 425 | 21.36 |  |
| Get Daily Newspaper | Yes | 69 | 3.47 | 40 | 2.01 | 109 | 5.48 | **<0.001*** |
|  | No | 648 | 32.56 | 1233 | 61.96 | 1881 | 94.52 |  |
| Ever pregnant | Yes | 459 | 23.07 | 1033 | 51.91 | 1492 | 74.97 | **<0.001*** |
|  | No | 258 | 12.96 | 240 | 12.06 | 498 | 25.03 |  |
| Ever used family planning | Yes | 717 | 36.03 | 827 | 41.56 | 1544 | 77.59 | **<0.001*** |
|  | No | 0 | 0.00 | 446 | 22.41 | 446 | 22.41 |  |
| Ever use OCP | Yes | 367 | 18.44 | 398 | 20.00 | 765 | 38.44 | **<0.001*** |
|  | No | 350 | 17.59 | 875 | 43.97 | 1225 | 61.56 |  |
| Currently using OCP | Yes | 357 | 17.94 | 129 | 6.48 | 486 | 24.42 | **<0.001*** |
|  | No | 360 | 18.09 | 1144 | 57.49 | 1504 | 75.58 |  |
| You involved in any NGO programs | Yes | 110 | 5.53 | 120 | 6.03 | 230 | 11.56 | **<0.001*** |
|  | No | 607 | 30.50 | 1153 | 57.94 | 1760 | 88.44 |  |

**S1 Table :** (Continued)

| Variables | Categories | Currently Using Family Planning  Yes No | | | | Total | | P Value |
| --- | --- | --- | --- | --- | --- | --- | --- | --- |
|  |  | n | % | n | % | N | % |  |
| No. of children | None | 178 | 8.94 | 250 | 12.56 | 428 | 21.51 |  |
|  | One | 259 | 13.02 | 207 | 10.40 | 466 | 23.42 |  |
|  | Two | 230 | 11.56 | 488 | 24.52 | 718 | 36.08 | **<0.001*** |
|  | Three | 30 | 1.51 | 248 | 12.46 | 278 | 13.97 |  |
|  | Four / Four+ | 20 | 1.01 | 80 | 4.02 | 100 | 5.03 |  |
|  | Have Childs, No More | 208 | 10.45 | 270 | 13.57 | 478 | 24.02 |  |
| No. of wanted child | One | 279 | 14.02 | 307 | 15.43 | 586 | 29.45 | **<0.001*** |
|  | Two | 200 | 10.05 | 598 | 30.05 | 798 | 40.10 |  |
|  | Three | 30 | 1.51 | 98 | 4.92 | 128 | 6.43 |  |
|  | None | 657 | 33.02 | 1163 | 58.44 | 1820 | 91.46 |  |
| No. of unwanted child | One | 50 | 2.51 | 100 | 5.03 | 150 | 7.54 | 0.338 |
|  | Two | 10 | 0.50 | 10 | 0.50 | 20 | 1.01 |  |
|  | None | 677 | 34.02 | 1003 | 50.40 | 1680 | 84.42 |  |
| No. of mistimed child | One | 40 | 2.01 | 160 | 8.04 | 200 | 10.05 | **<0.001*** |
|  | Two | 0 | 0.00 | 110 | 5.53 | 110 | 5.53 |  |
|  | None | 178 | 8.94 | 250 | 12.56 | 428 | 21.51 |  |
| No. of living children | 0 - 1 | 259 | 13.02 | 207 | 10.40 | 466 | 23.42 | **<0.001*** |
|  | 2 - 3 | 260 | 13.07 | 726 | 36.48 | 986 | 49.55 |  |
|  | 4 + | 20 | 1.01 | 90 | 4.52 | 110 | 5.53 |  |
|  | No Child | 178 | 8.94 | 260 | 13.07 | 438 | 22.01 |  |
| Child sex composition: | Only Daughter | 149 | 7.49 | 176 | 8.84 | 325 | 16.33 | **<0.001*** |
|  | Only Son | 170 | 8.54 | 200 | 10.05 | 370 | 18.59 |  |
|  | Both | 220 | 11.06 | 637 | 32.01 | 857 | 43.07 |  |
| Are you Pregnant now? | Yes | 63 | 3.17 | 116 | 5.83 | 179 | 8.99 | 0.807 |
|  | No | 654 | 32.86 | 1157 | 58.14 | 1811 | 91.01 |  |
| Ever conceived unexpectedly | Yes | 169 | 8.49 | 261 | 13.12 | 430 | 21.61 | 0.110 |
|  | No | 548 | 27.54 | 1012 | 50.85 | 1560 | 78.39 |  |
| Take the unwanted child | Never Happened | 537 | 26.98 | 1003 | 50.40 | 1540 | 77.39 |  |
|  | Yes | 133 | 6.68 | 227 | 11.41 | 360 | 18.09 | **0.004*** |
|  | No | 47 | 2.36 | 43 | 2.16 | 90 | 4.52 |  |
| Miscarriage/abortion Record | Yes | 38 | 1.91 | 42 | 2.11 | 80 | 4.02 | **0.029*** |
|  | No | 679 | 34.12 | 1231 | 61.86 | 1910 | 95.98 |  |
|  | Below 10 | 517 | 25.98 | 536 | 26.93 | 1053 | 52.91 |  |
| Duration of Marriage (Year) | 10 - 20 | 160 | 8.04 | 289 | 14.52 | 449 | 22.56 | **<0.001*** |
|  | Above 20 | 40 | 2.01 | 448 | 22.51 | 488 | 24.52 |  |

Chi-Square test was considered significant when the P Value found less than 0.05. All significant data were bolded.

**S1 Fig:** Preferred types of family planning method (Traditional) during COVID-19 pandemic

**S2 Fig:** Side effects of family planning methods during COVID-19 pandemic
