# Supplementary figures and images for: Prevalence and factors associated with family planning during COVID-19 pandemic in Bangladesh: A cross-sectional study
Source: PLoS One. 2021 Sep 21;16(9):e0257634. doi: 10.1371/journal.pone.0257634 (PMC8454962; doi:10.1371/journal.pone.0257634)

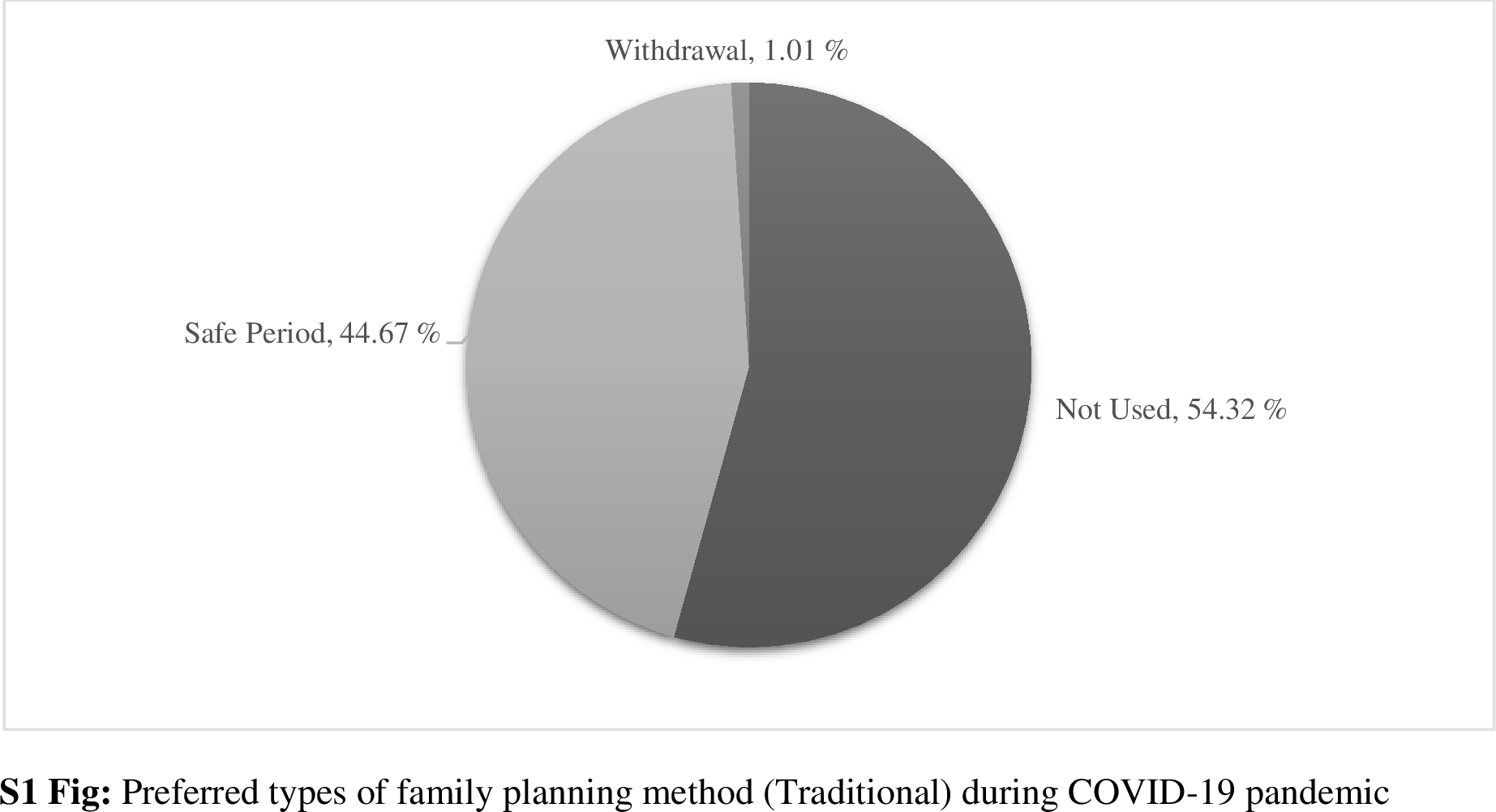

Supplement: S1 Fig — (TIF) [file pone.0257634.s004.tif]

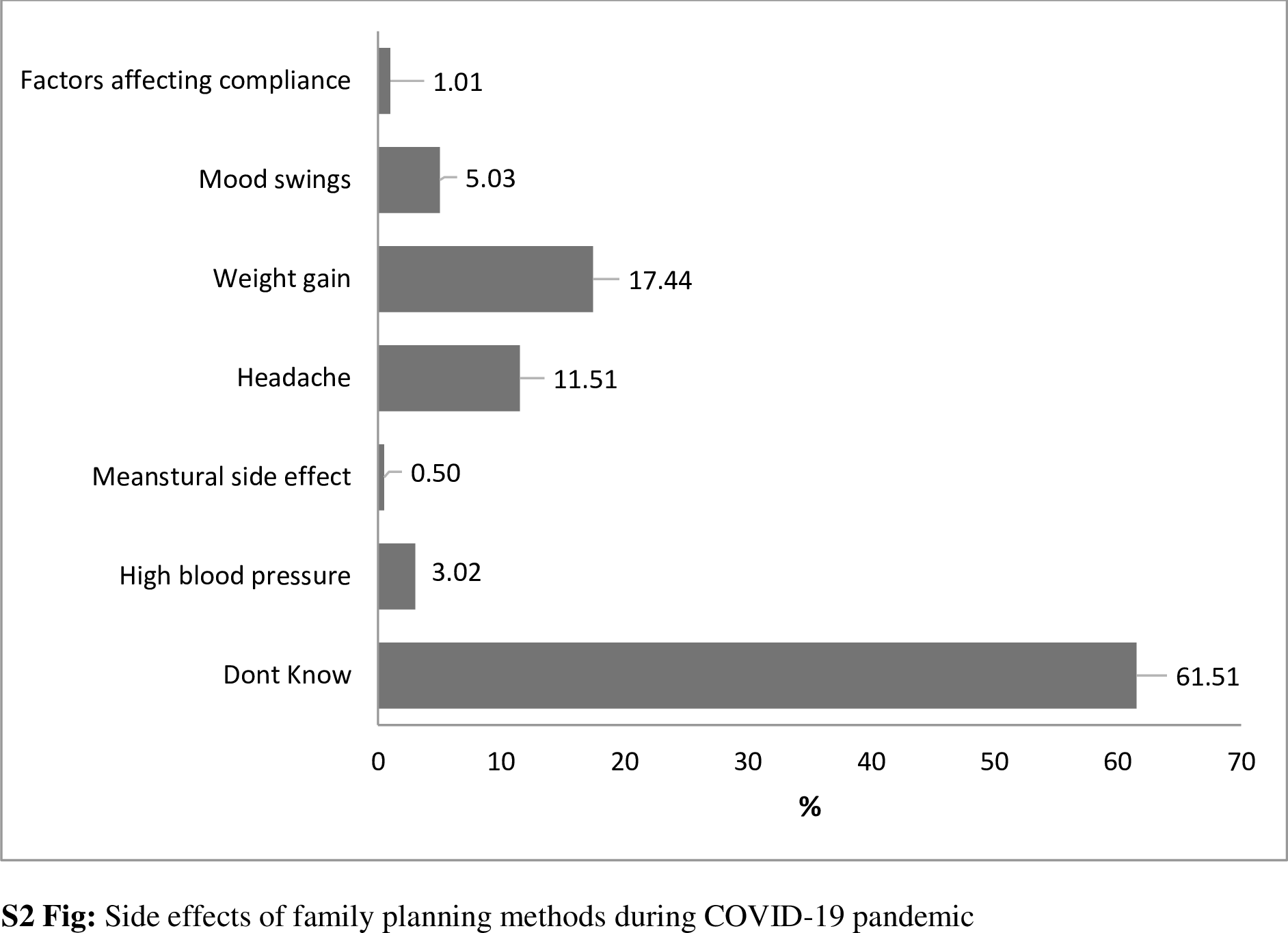

Supplement: S2 Fig — (TIF) [file pone.0257634.s005.tif]
